# Pediatric *Candida* Manifestations in the Orofacial Region: A Retrospective Analysis of Different Forms, Risk Factors and Species Distribution

**DOI:** 10.3390/jof11050363

**Published:** 2025-05-07

**Authors:** Sara Carina Kakoschke, Sara Fleschutz, Elisabeth Ruff, Karl Dichtl, Moritz Groeger, Carola Schoen, Sven Otto, Tamara Katharina Kakoschke

**Affiliations:** 1 Department of Oral and Maxillofacial Surgery and Facial Plastic Surgery, University Hospital, Ludwig-Maximilians-University Munich, Lindwurmstrasse 2a, 80337 Munich, Germany; 2 Department of General, Visceral, and Transplant Surgery, University Hospital, Ludwig-Maximilians-University Munich, Marchioninistrasse 15, 81337 Munich, Germany; 3 Max von Pettenkofer-Institut für Hygiene und Medizinische Mikrobiologie, Medizinische Fakultät, Ludwig-Maximilians-University Munich, Pettenkoferstraße 9A, 80336 Munich, Germany; 4 Diagnostic and Research Institute of Hygiene, Microbiology and Environmental Medicine, Medical University of Graz, Neue Stiftingtalstraße 6, 8010 Graz, Austria; 5 Department of Otorhinolaryngology, University Hospital, Ludwig-Maximilians-University Munich, Marchioninistrasse 15, 81337 Munich, Germany; 6 University Children’s Hospital, Dr. von Haunersches Kinderspital, Ludwig-Maximilians-University Munich, Lindwurmstraße 4, 80337 Munich, Germany

**Keywords:** *Candida*, oral candidiasis, head and neck infections, dental abscess, otitis media, middle ear, pediatric fungal infections, risk factors

## Abstract

The aim of this study was to analyze the spectrum of *Candida* manifestations in the orofacial region to address the lack of comprehensive data in the diverse pediatric population. This retrospective study included all positive *Candida* findings in patients aged 0–18 years treated between 2014 and 2023 at a university maxillofacial department in Germany and evaluated associated risk profiles, comorbidities and species distributions. *Candida* infection sites included oral mucosa, dental abscesses and otitis media. *Candida* was more frequent in children with pre-existing conditions, particularly immunosuppression, neuromuscular disorders and facial deformities. Tympanostomy tubes and recent antibiotic use were significant risk factors for *Candida* in otitis media. Whereas in dental abscesses, *Candida* had a significant proportion independent of prior antibiotic use. Non-*albicans* subspecies, particularly *Candida parapsilosis*, were notably more prevalent in the middle ear compared to oral and dental infections. *Candida* manifests in various forms in the orofacial region, with different characteristics and species distributions. Further investigations are needed to better understand the role of *Candida* as a symptom or a contributor to an underlying condition.

## 1. Introduction

Yeasts of the genus *Candida* colonize the skin and mucous membranes of many healthy individuals without having a pathogenic character [1,2,3,4]. If the composition of the skin or mucous flora becomes imbalanced, it can lead to a *Candida* infection. This is usually localized (e.g., oral or vaginal candidiasis), but in immunocompromised individuals, it can spread, become invasive or potentially even trigger candidemia, which is associated with high morbidity and mortality rates [5,6,7]. Patients with hematopoietic disorders and cancer are particularly affected by invasive candidiasis [8,9,10], which poses a significant challenge, especially in critically ill children [11]. *Candida albicans* is the most common agent, but the incidence of non-*albicans* infections is increasing [12,13,14]. *Candida parapsilosis* carries particular significance in pediatrics, especially neonatal candidemia, as it is frequently isolated from the bloodstream [15,16,17,18]. In recent years, also new *Candida* species were identified and increasingly discussed. For instance, *Candida auris* (first described in 2009) is feared in pediatrics due to its high rates of resistance and its potential for nosocomial outbreaks [19].

Due to its severity and the specific management in prevention and therapy [20], most publications address invasive candidiasis in children. There are limited research data available on local manifestations concerning young patients.

A common local infection that can also precede invasive candidiasis is oral candidiasis. It can manifest in various ways. The pseudomembranous form is thereby the most common. It is characterized by white, creamy patches on the soft tissue of the oral cavity (e.g., tongue, palate and buccal) and is commonly known as thrush. In addition to age (often newborns or elderly) and immune status (often immunocompromised individuals), risk factors include the oral administration of steroids (e.g., asthma sprays) or dry mouth [21,22]. There are acute and chronic forms of pseudomembranous candidiasis. Erythematous candidiasis can also occur acutely or chronically and causes redness and inflammation of the oral mucosa, which can result in a burning sensation. Affected areas of the oral mucosa are atrophic and sometimes associated with edema. The acute erythematous form is often preceded by antibiotic therapy, while the chronic form is usually associated with dentures [21,23,24]. Angular cheilitis often affects both corners of the mouth and causes cracks, redness and sometimes pain [21]. Another form of oral candidiasis is hyperplastic candidiasis, which presents as thickened, white grayish patches, often on the buccal mucosa, that cannot be easily whipped off compared to pseudomembranous candidiasis. It often occurs in smokers or individuals with poor oral hygiene, seldomly in children, and carries a potential precancerous risk [21,24]. Median rhomboid glossitis, also rare in children, is a benign lesion in the middle of the tongue that is associated with *Candida* [25]. The affected area is often smooth, flattened or slightly elevated and may appear red or leukoplakic [24,26].

Mucocutaneous candidiasis typically involves not only oral manifestations but also *Candida* infections in other body areas, such as the skin, nails, skin folds or genital region [27,28,29]. A rare form is chronic mucocutaneous candidiasis (CMC). It primarily affects patients with autoimmune diseases or congenital or acquired immune deficiencies. It leads to persistent or recurrent *Candida* infections, often based on mutations impairing cell-mediated immune defense against *Candida* [30,31,32].

There are more data available on the prevalence of oral *Candida* colonization than on oral *Candida* infections in children. Most studies have focused on children with underlying oncological conditions such as leukemia [33,34] or cancer [35]. Healthy preschool and school children have been identified as *Candida* carriers in 40–65% of cases [36,37]. Additionally, approximately half of all newborns and infants have been found to be colonized in the oral cavity [24,38]. *Candida* is believed to have cariogenic potential and to be associated with early childhood caries [39,40,41].

*Candida* spp. have also been isolated in endodontic and periodontal infections, as well as in association with dental implants [42,43,44,45]. Therefore, it is not surprising that *Candida* spp. can be found in 3–8% of dental (odontogenic) abscesses in adults [46,47,48,49,50,51,52,53].

The literature also describes non-odontogenic facial (skin) abscesses and extensive facial infections caused by *Candida* [54,55,56,57,58,59,60]. Other manifestations of *Candida* infections in the facial area can involve the eyes [61,62] and tear ducts [60,63,64] or the ears.

Otomycoses can affect various parts of the ear (such as the external auditory canal, middle ear and inner ear) and are most commonly caused by *Candida* or *Aspergillus. Candida* infections are usually more difficult to recognize, as they do not show as characteristic morphological features as *Aspergillus* infections but rather present as purulent infections or otorrhea with no response to antibiotics [65]. Otitis externa and media are common pediatric conditions. *Candida* spp. can also be isolated in immunocompetent children, and an increase in otomycoses has been observed recently [66].

Despite the need for comprehensive data on *Candida* infections of the orofacial region in children, especially when considering beyond individual pediatric subgroups, there is a scarcity of such information available. This study aims to reduce this deficit. Here, all children (age 0–18 years) who presented at a university center for oral and maxillofacial surgery over a period of nearly 10 years were retrospectively analyzed and filtered for microbiological findings involving *Candida*. We identified various *Candida* manifestations of the oral mucosa, dental abscesses and middle ear and placed a special focus on the spectrum of different *Candida* species and risk factors.

## 2. Materials and Methods

This monocentric, explorative, retrospective study was approved by the LMU Ethics Committee (protocol code: 23-0344; date of approval: 26 May 2023).

The clinical and demographic data of pediatric patients aged 0–18 years, who received outpatient or inpatient treatment at the Department of Oral and Maxillofacial Surgery at LMU Hospital between January 2014 and March 2023, were collected and analyzed. Among them, patients with samples that were culture-positive for *Candida* were identified and separately evaluated.

Identification was performed from overnight cultures of *Candida* (incubation at 35 ± 1 °C on Sabouraud, blood or chocolate agar) using a Bruker Biotyper smart MALDI-TOF MS device (Bruker Daltonics, Bremen, Germany) with the respective current databases for the periods under investigation.

The location(s) of the fungus, type of infection, clinical findings, results of species differentiation, underlying and pre-existing conditions, comorbidities and risk factors were extracted from the patient records.

The type of *Candida* manifestation was determined based on the documented diagnosis and clinical findings. Infections that lasted for more than six weeks were classified as chronic. Asymptomatic colonization referred to those who had evidence of *Candida* presence without clinical signs of infection. Mucocutaneous candidiasis included those who had oral candidiasis and at least one additional infection on the skin (and possibly other mucous membranes) that was/were located outside the oral area.

Exclusion criteria were incomplete access or availability of the documentation. The data were anonymized before analysis. Demographic, epidemiological and clinical outcomes were evaluated and presented using descriptive statistics. To investigate whether the detection of *Candida* in dental abscesses or otitis media was associated with the presence of risk factors or recent/frequent antibiotic use, the Fisher’s Exact Test was applied. The result was considered significant at a *p*-value < 0.05. For the calculations and presentation of the results, GraphPad Prism Version 9.5.1 was used.

## 3. Results

Out of 10,828 examined children, 1191 had microbiological swab results from the orofacial region. Among these, 134 (11%) children were tested positive for *Candida* in the head and neck region. These *Candida*-positive swabs were taken from the oral mucosa (75 children), pus from dental abscesses (40 children) or discharge from the middle ear (19 children). The middle ear swabs were exclusively obtained from children with facial clefts. The gender distribution was balanced in all groups, with no significant differences observed. There was no invasive or septic event recorded.

### 3.1. Oral Candidiasis

Twenty-eight children were documented with microbiologically proven oral candidiasis (OC). Among them, 15 had pseudomembranous candidiasis (10 acute, 5 chronic), 2 had acute erythematous candidiasis, 9 had mucocutaneous candidiasis (all chronic), and 2 had acute forms of angular cheilitis (Table 1).

**Figure 1:**
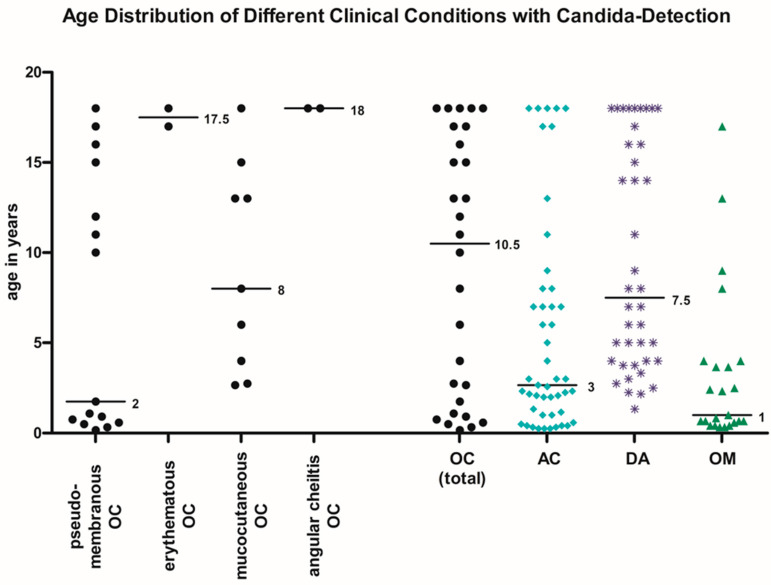
Age distribution of different orofacial *Candida* manifestations (individual values and corresponding median are plotted for the different clinical pictures).

The age distribution is shown in Figure 1. Half of the children with pseudomembranous candidiasis were under 2 years old, while the other half were 10 to 18 years old. The erythematous form and angular cheilitis were found near adulthood, with both cases each being around 18 years. The mucocutaneous candidiasis showed a broader age distribution, with a median age of 8 years. Half of the children were in preschool or primary school age, while the other half were teenagers. When considering all forms of OC together, manifestations were observed in almost every age group. However, cases were more frequent in infants and toddlers, as well as in late adolescence.

Focusing on the locations where *Candida* was isolated, it is notable that more than 64% of the children with OC also had positive *Candida* detections outside the oral cavity (Table 1). To distinguish the infections from asymptomatic colonization, the infection sites are marked in gray in Table 1.

Intraorally, the majority of positive samples for all forms of OC were taken from the tongue (n = 19/67.9%), followed by the palate (n = 13/46.4%) and buccal mucosa (n = 6/21.4%) (Table 1).

A total of 92.9% of children with OC had comorbidities and risk factors (Table 2). Immunological disorders, neurological or neuromuscular disorders and facial deformities such as congenital cleft lip and palate were the most prevalent comorbidities. Three patients had macroglossia.

Nine children with OC had *Candida* infections also in other areas of the body and were therefore classified as mucocutaneous candidiasis. All of them were chronic forms, persisting longer than 6 weeks. All children with mucocutaneous candidiasis had multiple comorbidities, and a majority (five out of nine patients) had genetic disorders affecting the immune system, such as XIAP-like immunoregulatory syndrome and combined immunodeficiencies or chromosomal aberrations. Three had autoimmune diseases. Typical gene mutations attributed to CMC, such as defects in STAT genes, IL genes, AIRE or CARD9 [32], were not found or documented.

A total of 7 of 28 children (25%) with OC had a percutaneous endoscopic gastrostomy (PEG). All seven had positive *Candida* cultures in the PEG area, and out of these, five had manifest *Candida* infection of the PEG region.

### 3.2. Asymptomatic Oral Candida Colonization

Forty-seven children were found to have asymptomatic oral *Candida* colonization (AC) of the oral cavity without any symptoms.

Half of all children with AC were under 3 years old, with the majority being infants. However, all age groups were represented (Figure 1). A total of 80.9% of AC had comorbidities (Table 2). In contrast to OC, there were fewer immunological disorders. Alongside neurological and neuromuscular disorders, a significant portion of patients had chronic lung diseases requiring the use of corticosteroid inhalation sprays. Congenital facial deformities accounted for the majority of diagnoses (Table 2). Macroglossia was known in six cases.

Unlike OC, the exact location of the intraoral swab was often not specified in the medical records. However, a significant proportion of extraoral co-localizations were observed as well (Table 3).

### 3.3. Dental Abscesses and Candida Detection

During the study period, a total of 821 children presented with dental (odontogenic) abscesses (DAs). Swabs were taken from 272 children with DAs, and 40 of these patients (14.7%) showed evidence of *Candida* within the pus of DAs.

**Figure 2:**
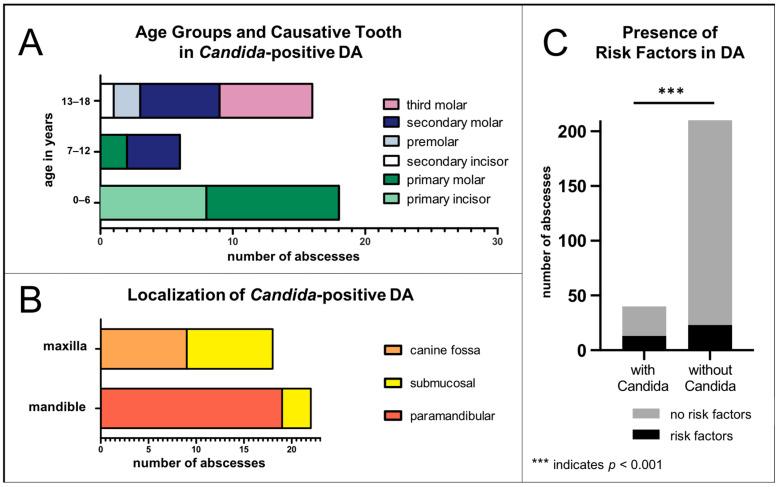
Dental abscesses (DAs) with *Candida* detection: (**A**) age groups and causative tooth of DA with *Candida*; (**B**) localization of DA with *Candida*; (**C**) comparison of the prevalence of comorbidities/risk factors in DA with and without *Candida*.

The median age of the children with *Candida*-positive DAs was 7.5 years (Figure 1). There was also a second peak around the age of 18. The cause of the DA in all cases was caries except when the causative tooth was a wisdom tooth. Then it was due to tooth eruption disorders and pericoronitis. The causative tooth in relation to the age group is shown in Figure 2A. In the age group 0–6 years, children are usually in their primary dentition. In the age group 7–12 years, they are in the mixed dentition phase. In the age group 13–18, they are typically in their permanent dentition (after the eruption of the 12-year molar). Wisdom teeth or third molars can start erupting during the teenage years.

There were slightly more abscesses located in the lower jaw (n = 22) than in the upper jaw (n = 18) (Figure 2B). The proportion of strictly localized submucosal abscesses was higher in the upper jaw (n = 9) than in the lower jaw (n = 3). In the lower jaw, most abscesses were located in the paramandibular space (n = 19), while in the upper jaw, they often extended into the canine fossa (n = 9) (Figure 2B). However, no abscess showed a tendency for extensive spread.

Children with *Candida* in the abscess had a significantly lower proportion of comorbidities or risk factors, with 32.5%, compared to the group with OC or AC (92.9% and 80.9%, respectively *p* < 0.0001) (Table 2): Two children were undergoing chemotherapy or stem cell transplantation, three children had heart defects, and another three had asthma bronchiale. One had a repaired cleft palate, one had hemophilia, one had diabetes, and two had epilepsy (one with cerebral palsy). However, 67.5% of the children with DAs were healthy. Nevertheless, compared to children without *Candida* in the abscess, the proportion of those with risk factors was significantly higher, when *Candida* was found in the abscess (Figure 2C). A total of 32.5% of the children with *Candida*-positive DAs had risk factors, while only 9.9% of the children with *Candida*-negative DAs had risk factors (*p* = 0.0005).

### 3.4. Otitis Media in Cleft Patients

Children with palatal clefts can have reduced middle ear ventilation due to incorrect insertion of the palate muscles, which often prevents the Eustachian tube from fully opening [67]. This can lead to effusions, causing dampened sound conduction with hearing impairment and potential infections.

During the study period, a total of 1203 children with facial clefts presented to our department for oral and maxillofacial surgery. Within this group, 142 swabs were collected from 112 children with clefts and otitis media with effusion (OMs). *Candida* was detected in the exudate of 23 swabs (16.2%) from 19 children (16.9%).

What stands out in the OM group is that the children positive for *Candida* had no other comorbidities except facial deformities. More than half of the children were under one year old. At our department for oral and maxillofacial surgery, primary surgeries for cleft lip and palate (lip repair, palatoplasty) are typically performed within the first year of life. Our ENT colleagues join the surgeries for ear tube insertion when indicated. After successful palate closure, the frequency of OMs usually decreases over time when the misinsertion of the palatal muscles has been corrected. However, the interval for middle ear improvement varies individually. The children attend regular ENT follow-up appointments during which tympanostomy tubes are monitored and, if necessary, replaced, removed or reinserted. In all cases where *Candida* was detected, tympanostomy tubes were already in place at the time of swab collection. In comparison, 70.9% of the children without *Candida* had tympanostomy tubes in place. Tympanostomy tubes could therefore represent a risk factor for *Candida* colonization of the middle ear (*p* = 0.006).

### 3.5. Microbiological Candida Spectrum

**Figure 3:**
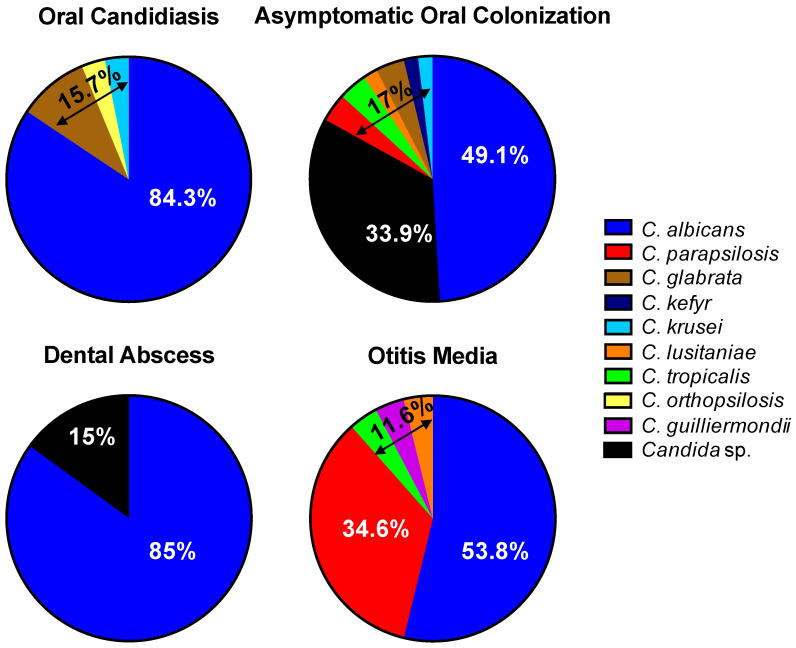
Spectrum of different *Candida* isolates in the four different orofacial manifestations: oral candidiasis, asymptomatic oral colonization, dental abscess and otitis media. No species differentiation was performed in the cases reported with the result “*Candida* sp.”.

When examining the microbiological spectrum of *Candida* in our four subgroups with *Candida* manifestations in the head and neck area, differences became apparent (Figure 3). In the group with OMs, *Candida albicans* was detected in 53.8% of cases, *Candida parapsilosis* in 34.6% of cases and *Candida tropicalis*, *Candida guilliermondii* and *Candida lusitaniae* in the remaining 11.7% of cases. The proportion of non-*albicans* strains was significantly higher in middle ear manifestations (46.2%) compared to oral manifestations, with *Candida parapsilosis* clearly leading the non-*albicans* strains in OMs. The most diverse range of *Candida* spp. was found in AC, where *Candida albicans*, *Candida parapsilosis*, *Candida tropicalis*, *Candida lusitaniae*, *Candida glabrata*, *Candida kefyr* and *Candida krusei* were detected. However, in 33.9% of cases, the finding was reported as “*Candida* sp.” without further differentiation.

The microbiological spectrum of DAs was very similar to the one of OC. The proportion of *C. albicans* was 85% in DAs and 84.3% in OC. In OC *Candida glabrata*, *Candida krusei* and *Candida orthopsilosis* were found.

Apart from intrinsic resistances, no acquired resistances against common antifungal agents were observed in this study.

In OC, 42.8% and in AC, 36.1% of the children had a history of previous or frequent systemic antibiotic use. In OMs, the proportion was even higher: In 86.9% of ear swabs with *Candida*, the children had recently received systemic antibiotics, and in 65.2% of cases, they had previously applied local antibiotic-containing ear drops. In ear swabs without *Candida*, 30.2% had received systemic antibiotics, and 12.6% had used local antibiotic-containing ear drops. This indicates a significant association between prior antibiotic use and the presence of *Candida* in middle ear effusions (*p* < 0.0001). In *Candida*-positive DAs, only 5% received antibiotics prior drainage, and no significant differences were found in the DA groups regarding recent antibiotic use.

## 4. Discussion

This is a retrospective study comparing the characteristics of pediatric patients with and without *Candida* in infected sites of the orofacial region. Oral candidiasis (OC) and its subtypes were examined as well as asymptomatic *Candida* colonization (AC) of the oral cavity without signs of infection. For *Candida*-positive cases, additional finding of *Candida* outside of the orofacial region was also documented. Moreover, *Candida* was analyzed in dental abscesses (DAs) and otitis media (OMs). No clear distinction between colonization and infection could be made in cases of *Candida* detection in DAs and OMs. As the data were based on retrospective culture findings without histopathologic confirmation or detailed clinical correlation, the clinical significance of *Candida* in these settings remains uncertain. Consequently, we interpret these findings cautiously and primarily as colonization unless infection signs were explicitly documented.

Due to the heterogeneity of patient profiles and lack of defined comparison groups for the OC and AC cohorts, further statistical testing beyond descriptive analysis was not feasible for OC and AC. However, for the DA and OM groups, children with and without *Candida* detection could be directly compared providing important information that is rarely or not found in the literature so far. We provided statistical analysis comparing risk factors in the DA and OM groups. Additional subgroup analysis, such as species-specific patterns or age stratification within *Candida*-positive DAs/OMs, was considered but limited by sample size.

We found that *Candida* was detected in nearly 15% of children with DAs, which at first seems surprisingly high but has not yet been studied in children before. In adults, the proportion of *Candida* in DAs in previous studies was significantly lower, being 3–8% [46,47,48,49,50,51,52,53]. Strikingly, the *Candida*-positive children in the DA group had significantly higher rates of comorbidities, even though the majority was healthy overall. Although the proportion of *Candida* in DAs and OMs was similarly high in our study (DA: ca. 15%, OM: ca. 16%), the results suggest that frequent or recent antibiotic use does not represent a risk factor for *Candida* in DAs, but it does for OMs.

Notably, antibiotic exposure is a well established risk factor for *Candida* colonization and infection, as it disrupts the normal bacterial microbiota and reduces microbial competition, thereby promoting fungal overgrowth. This effect has been documented in the context of oral candidiasis and otomycosis [29,68]. Our data support this association, particularly in the OM group, where prior antibiotic use showed a significant correlation with *Candida* detection.

Additionally, our results show that *Candida* was significantly more common in cases where tympanostomy tubes were already in place compared to cases of OMs without tympanostomy tubes, suggesting that the tubes might be a risk factor for *Candida* colonization of the middle ear.

We evaluated four different entities of clinically manifest OC. As expected, oral pseudomembranous candidiasis was the most common, but a high proportion of mucocutaneous manifestations additionally affecting other parts of the body were also observed in our cohort. The latter mainly occurred in immunodeficient or immunodysregulated patients. This high proportion of mucocutaneous manifestations may be attributed to the overrepresentation of children with rare diseases in our university hospital setting compared to peripheral medical centers.

At our department for oral and maxillofacial surgery, we also have a higher proportion of children with orofacial clefts and other congenital or acquired deformities of the face including mouth and jaw (e.g., due to trauma or tumors). Therefore, it is not surprising that children with facial deformities are more frequently included in this study. However, it is already known in the literature that the oral flora in cleft patients exhibits peculiarities [69]. Thus, clefts have been identified as a risk factor for *Candida* colonization [70], and infants with cleft lip and palate show significantly higher *Candida* colonization rates compared to children without clefts [71,72], especially when functional orthodontic appliances or feeding plates are used [73]. The higher rate of colonization may be due to anatomical and physiological characteristics, as well as higher hospitalization rates and the frequent use of antibiotics associated with multiple surgical interventions. The proportion of *Candida*-positive OMs was over 16% in our cleft patients. There are data suggesting that *Candida* is more frequently recovered in OMs [74], but further investigations are needed to determine whether the microbiological spectrum of OMs differs between cleft patients and non-cleft patients.

Considering other risk factors, our study also reveals a high proportion of neurological and neuromuscular disorders in children with OC and AC. Motoric dysfunctions can lead to impaired physiological self-cleaning abilities of the oral cavity. Oral hygiene can also be hindered by limited motoric and/or cognitive compliance [75]. We also observed some children with macroglossia among oral *Candida* manifestations (three in OC and six in AC, Table 2). Macroglossia can result in restricted tongue movement due to limited space in the oral cavity, potentially affecting self-cleansing functions. However, an enlarged tongue can also impact feeding, dental alignment and breathing. Children with macroglossia often do not close their mouths fully or frequently, allowing more space for the tongue. All of these factors could contribute to an altered oral flora. However, there are limited data available on macroglossia and oral microbiological composition. Some studies have examined children with Down syndrome [76,77], but these children often have additional risk factors alongside macroglossia, making it difficult to assess the influence of only macroglossia in this regard. Also, in our study, macroglossia was associated with other clinical findings.

Another risk factor has already been investigated in international studies and is consistent with our data: The proportion of patients with chronic lung diseases and frequent inhalation of corticosteroids is also increased among our AC and DA patients [78,79].

From a clinical perspective, preventive strategies in high-risk pediatric populations should focus on minimizing modifiable risk factors. These include judicious use of systemic antibiotics, promoting oral hygiene and consistent care of medical devices such as PEG tubes. Children with immunosuppression or neuromuscular disorders may benefit from regular oral screenings to detect early signs of *Candida* colonization or infection. For treatment, topical antifungals such as nystatin or miconazole are recommended for localized oral candidiasis [22,27], while systemic antifungals (e.g., fluconazole) may be necessary in cases of mucocutaneous involvement or in immunocompromised hosts [7]. Prophylactic antifungal treatment is not routinely indicated in non-severely immunosuppressed children but may be considered on a case-by-case basis in high-risk oncology or transplant settings [20].

Regarding the age distribution, it is noticeable that *Candida* was predominantly detected in patients in early childhood. The average age in the subgroup of *Candida*-positive DAs was slightly higher. However, this can be explained by the fact that DAs mainly occur in the primary dentition of children with an average age of 5–6 years [80,81]. Whether *Candida* colonization of the oral cavity actually decreases after early childhood and increases again in adulthood would need to be investigated in longitudinal studies.

Our retrospective data show that the proportion of *C. albicans* in OC as well as DAs is approximately equal but differs from those of the middle ear. In our OM group, the proportion of non-*albicans* strains, especially *C. parapsilosis*, was significantly higher.

Nowadays, there are sensitive methods available, such as microbiome analyses, that could capture precise differences in the spectrum of *Candida* species of the oral and ear flora. It remains to be investigated whether *Candida* is simply an opportunistic colonizer in risk patients or if it has a pathogenic influence in certain clinical conditions.

## 5. Conclusions

In summary, this retrospective study highlights the heterogeneity of *Candida* manifestations in the pediatric orofacial region. *Candida* species were detected not only in classic oral candidiasis but also in dental abscesses and middle ear effusions. The findings underscore the importance of tailored diagnostic and therapeutic approaches and indicate the need for further research to delineate the pathogenic versus colonizing role of *Candida* in different orofacial sites.

## Figures and Tables

**Table 1:** Oral candidiasis: localization of *Candida* infections of the oral mucosa detected via swab and, if diagnosed, additional specimens and sampling sites positive for *Candida*. Subdivision into the identified subtypes of oral candidiasis. The sites with clinical signs of infection are marked in gray to distinguish them from *Candida* findings without signs of infection (presumably colonization).

		**Oral Candidiasis**																											
		**Pseudomembraneous**															**Erythematous**		**Mucocutaneous**									**Angular Cheilitis**	
	**Total**	**1**	**2**	**3**	**4**	**5**	**6**	**7**	**8**	**9**	**10**	**11**	**12**	**13**	**14**	**15**	**16**	**17**	**18**	**19**	**20**	**21**	**22**	**23**	**24**	**25**	**26**	**27**	**28**
Oral mucosa	28	x ^a^	x ^a^	x ^a^	x ^a^	x ^a^	x ^c^	x ^c^	x ^c^	x ^a^	x ^a^	x ^a^	x ^a^	x ^a^	x ^c^	x ^c^	x ^a^	x ^a^	x ^c^	x ^c^	x ^c^	x ^c^	x ^c^	x ^c^	x ^c^	x ^c^	x ^c^	x ^a^	x ^a^
- Buccal	6	x				x													x			x			x				x
- Tongue	19		x	x	x		x	x	x	x	x	x			x	x	x	x	x	x	x			x	x		x		
- Palatine	13		x			x	x		x		x		x	x			x		x	x	x		x			x			
- Lips	3																		x									x	x
Other sites *	18	x	x				x		x	x				x	x	x			x	x	x	x	x	x	x	x	x		x
- Sputum	4																					x		x		x			x
- Pharyngeal	3						x		x							x													
- Tracheal	7						x		x	x				x	x						x	x							
- Esophageal	1															x													
- Nose	4		x							x												x	x						
- Ear	1	x																											
- Eye	1													x															
- Anal	4								x											x	x					x			
- Vaginal	3																		x		x			x					
- Inguinal	4																			x	x	x			x				
- Nails	2																							x			x		
- Skin	11								x						x				x	x	x	x	x	x	x	x	x		
- Urine	1																								x				
- PEG	7														x					x			x	x	x	x	x		

* Other detection sites except the oral mucosa; a = acute; c = chronic (>6 weeks).

**Table 2:** Risk factors and comorbidities of children with *Candida*-positive samples.

	Oral Candidiasis n = 28	Asymptomatic Colonization n = 47	Dental Abscess n = 40	Otitis media n = 19
No risk factors	2 (7.1%)	9 (19.1%)	27 (67.5%)	0
Risk Factors	26 (92.9%)	36 (80.9%)	13 (32.5%)	19 (100%)
Type of risk factors (multiple entries per child possible):				
Immunological disorders or malignancies	11	3	2	0
- Congenital immunodeficiencies	3	-	-	-
- Stem cell transplantation	2	-	1	-
- Liver transplantation	2	-	-	-
- Chemotherapy	-	1	1	-
- Autoimmune disease	3	-	-	-
- Hematooncological malignancy	1	-	2	-
- Solid cancer	-	2	-	-
Facial deformities	14	25	1	19
- Cleft lip and/or palate	9	17	1	19
- Others (congenital)	1	3	-	-
- Others (acquired)	1	5	-	-
- Macroglossia	3	6	-	2
Neurological or neuromuscular disorders	15	7	2	0
- Primary muscular dystrophy	2	3	-	-
- Muscular hypotony or myopathy	5	2	-	-
- Facial paralysis	-	1	-	-
- Cerebral palsy	3	1	1	-
- Neurodegenerative disease	2	1	-	-
- Epilepsy	3	2	2	-
Lung disease	0	8	3	0
- Asthma bronchiale/COPD	-	4	3	-
- Cystic fibrosis/primary ciliary dyskinesia	-	4	-	-
Hemophilia	0	0	1	0
Metabolic disorders	2	1	1	0
- Diabetes mellitus	1	-	1	-
- Storage disorders	2	1	-	-
Cardiovascular disease	1	2	3	0
Chronic skin disease	4	0	0	0
Chromosomal aberrations	5	3	1	0
PEG (percutaneous endoscopic gastrostomy)	7	8	0	0

**Table 3:** Oral asymptomatic colonization and other sites of *Candida* isolation except the oral mucosa.

	**Asymptomatic Oral Colonization**																																															
	**Total**	**1**	**2**	**3**	**4**	**5**	**6**	**7**	**8**	**9**	**10**	**11**	**12**	**13**	**14**	**15**	**16**	**17**	**18**	**19**	**20**	**21**	**22**	**23**	**24**	**25**	**26**	**27**	**28**	**29**	**30**	**31**	**32**	**33**	**34**	**35**	**36**	**37**	**38**	**39**	**40**	**41**	**42**	**43**	**44**	**45**	**46**	**47**
Oral mucosa	47	x	x	x	x	x	x	x	x	x	x	x	x	x	x	x	x	x	x	x	x	x	x	x	x	x	x	x	x	x	x	x	x	x	x	x	x	x	x	x	x	x	x	x	x	x	x	x
Other sites *	27	x	x		x	x	x	x	x	x						x	x		x	x	x		x			x	x	x	x	x	x	x		x	x	x	x	x	x									
- Sputum	3																						x											x	x													
- Pharyngeal	8								x														x			x	x	x								x	x	x										
- Tracheal	17		x		x	x	x	x		x						x	x		x	x	x		x						x	x	x	x		x														
- Esophageal	0																																															
- Nose	6	x											x		x									x									x						x									
- Ear	1	x																																														
- Eye	0																																															
- Anal	2																					x																	x									
- Genital	0																																															
- Inguinal	0																																															
- Nails	1							x																																								
- Skin	1							x																																								
- Urine	2			x																			x																									
- PEG	5	x									x			x					x			x																										

* Other detection sites except for the oral mucosa.

## Data Availability

The original contributions presented in this study are included in the article. Further inquiries can be directed to the corresponding author.
